# Simultaneous determination of 7 thiols associated proteins in lymphoma patients’serum and cerebrospinal fluid by UHPLC-HRMS technique

**DOI:** 10.1038/s41598-025-03023-6

**Published:** 2025-07-03

**Authors:** Qingkun Ma, Mei Zhang, Yongshuai Fan, Han Zhao, Kun Wang, Xiaojing Wang, Yanling Mu

**Affiliations:** 1 School of Pharmaceutical Sciences, Institute of Materia, National Key Laboratory of Advanced Drug Delivery System, Key Laboratory for Biotechnology Drugs of National Health Commission, Department of Pharmacy, Shandong First Medical University, Shandong Academy of Medical Sciences, Shandong Academy of Medical Sciences, the First Affiliated Hospital of Shandong First Medical University & Shandong Provincial Qianfoshan Hospital, Jinan, 250117 Shandong China; 2 Department of Neurosurgery Qingdao Huangdao District People’s Hospital, Qingdao, 266400 Shandong China

**Keywords:** Thiols related proteins, Br-OTPP, PCNSL, Machine learning, UHPLC orbitrap eclipse HRMS, Diagnostic markers, Predictive markers, Metabolomics, Mass spectrometry, Metabolomics

## Abstract

Thiol compounds can serve as markers for the antioxidant and prognostic status of lymphoma, playing a crucial role in early tumor diagnosis. However, their high polarity and lack of chromophores pose challenges for multivariate analysis. This study aims to measure thiols associated proteins and develop novel diagnostic models in serum and cerebrospinal fluid from PCNSL to verify potential association and early warning effectiveness. A highly sensitive and selective method was established for simultaneous determination of 7 different thiols and 340 related proteins based on self-developed mass spectrometry probe, Br-OTPP labeled by UHPLC-HRMS. Furthermore, a novel PCNSL monitoring model was developed based on different those combined with machine learning algorithm. Thiols has good linear relationship with correlation coefficients ≥ 0.9995 and suitable precision with inter-day and intra-day coefficients variation was 2.08–5.49% and 1.58–5.53%. Satisfactory accuracy with recoveries between 85.28 and 97.88% was observed. The limit of detection (S/*N* = 3) was 0.8-9.0 fmol. Ultimately, this method greatly improves discrimination efficiency of model with accuracy of 98.83%. The proposed method could successfully apply into measurement of thiols associated proteins and development of multi-omics clinical evaluation model for PCNSL patients.

**Supplementary Information:**

The online version contains supplementary material available at 10.1038/s41598-025-03023-6.

## Introduction

 PCNSL is a type of highly invasive non-Hodgkin’s lymphoma originating in the central nervous system^1,2^. 30% patients will have tumor recurrence or death within two years due to its high recurrence rate, poor prognosis and lacks specific clinical manifestations lead to easily misdiagnosed: (i). Enhanced magnetic resonance imaging, the phenotype of this examination is not specific due to the distinguish of glioma, intracranial infection and non-infectious inflammation difficulty^3^. (ii). Pathological examination, biopsy or resection of lesions is risky, some patients may have false negative results because of the deep location of the lesion, lack of tissue, or use of hormones^4^. (iii). Cellular immunopathology, this test requires a needle biopsy to obtain a specimen and the amount of tissue is limited^5^. Moreover, the occurrence of lymphoma was a multi-factor and multi-stage complex biological process. The detection of a single clinical index just identify tumor-related biomarkers at a single level and failed to understand the whole process of PCNSL occurrence^6^. Therefore, integrating proteomics and metabonomic are expected to find the abnormality of downstream metabolic network caused by genetic and environmental factors^7^. However, the current research is limited to the exploration of causal chain of “genetic/environmental factors-biomarkers-tumorigenesis”, Additionally, the exploration results of genetic/environmental factors have become increasingly mature at cellular level, but screening and verification of biomarkers in PCNSL patient’ serum and cerebrospinal fluid are still in progress^8,9^. Given this, this study of metabonomic in PCNSL patient’ cerebrospinal fluid would be helpful to reveal the complex interaction and etiology network of related protein and further reveal pathogenesis of PCNSL.

**Fig. 1:**
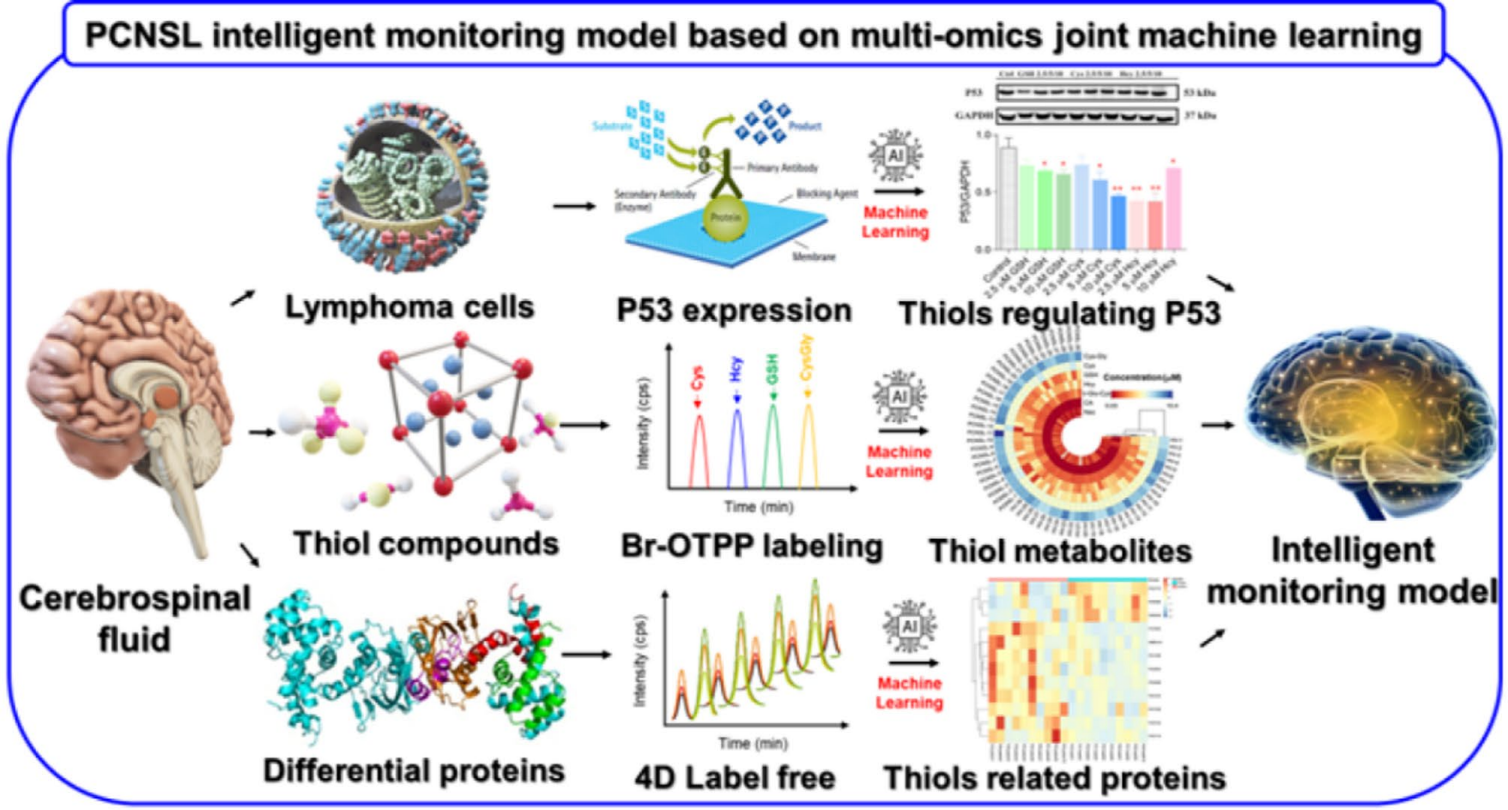
Intelligent monitoring model for primary central nervous system lymphoma (PCNSL) based on thiols associated proteins combined with machine learning.

Abnormal metabolism was one of the core markers of malignant tumor^10^. Among them, metabonomic of thiols can not only effectively identify lymphoma biomarkers, but also comprehensively reveal tumor-related metabolic network disorders, contributing to indepth understanding of the pathogenesis of lymphoma^11^. For instance, J Kovár et al. found that both Cys deprivation and overdose can induce apoptosis of lymphoma cells. Cys deprivation induced apoptosis was particularly associated with the presence of free SH groups^12^. Laurie Bruzzese illustrated that Hcy levels are associated with lymphocyte-mediated inflammatory responses^13^. Roberto Locigno suggested that the levels of GSH in lymphoma cells are significantly decreased^14^. In previous study, we found that the expression of p53 protein decreased with increase of thiol concentration in PCNSL cells as shown in Fig. 1. In addition, reports of thiols metabonomics and upstream differentially expressed proteins in PCNSL patients’ cerebrospinal fluid and serum was barely and unknown, respectively. Therefore, the development of a high-sensitive analytical methods for discovery of appropriate thiol biomarkers that may contribute to in-depth understanding of the pathogenesis and routine clinical diagnosis of PCNSL.

Thiol compounds have high polarity and no chromophore lead to traditional clinical detection techniques failed to meet new requirements of precision, comprehensive and timely precision medicine^15^. On one hand, spectroscopy and immunoabutology have weakness of single qualitative and quantitative characteristic information. On the other hand, the parallel and complementary drive of MS and NGS were cornerstone of regional personalized precision medicine^16^. But MS has ability to move from disease prediction to disease monitoring compared with NGS^17^. Moreover, the combination of superior separation ability of UHPLC and high specificity of MS could provide multidimensional characteristics of thiols^18-23^.

The feasibility and utility of thiol isolation through fluorescence/ultraviolet detection is profoundly influenced by the inventive chiral derivatization strategy introduced by Dongri Jin and Toshimasa Toyo’oka. This tactic uses an agent, DBD-PyNCS, to recognize targeted molecules for effective isolation^24^. Additionally, this reagent was utilized to analyze the levels of thiols in both plasma and urine. LOD for this method ranged from 0.4 to 2.4 pmol. In addition, the (S) -Naproxen-benzotriazole that has been developed was used as a derivative reagent by Ravi Bhushan and Rituraj Dubey to form Cys and Hcy diastereomers and achieve chiral separation by HPLC-UV with LOD of 0.0001–0.0015 nmol^25^. Furthermore, Sano et al. developed a derivatization method based on the combination of OPA and L-amino acids to measure Cys and Hcy simultaneously by HPLC-FL, achieving a detection limit of about a few picomoles^26^. In addition, Ito et al. accomplished the separation of the enantiomers of Cys and Hcy using HPLC-UV and achieved a LOD of 5 ng by employing derivatization with GITC^27^. Additionally, N-succinimide-(S)−2- (6-methoxy-naphthalene 2-ethyl) propionic acid was developed by Ravi Bhasan and Shivani Tanwar as labeled agent, labeled Pen by HPLC-UV with LOD of 0.5–2.5 pmol^28^. Moreover, Liu Ping presented an innovative technique for isotope labeling (d0/d7-BQB, specifically ω-bromoacetonylquinol inium-d0/d7-bromide). This approach was combined with high–performance liquid chromatography-double precursor ion scan mass spectrometry analysis to enable the nontargeted profiling of thiols. The limit of detection was ranged from 0.012 to 5.9 nM^29^. Our team previously developed a unique MS probe known as NCS-OTPP. This probe utilizes TPP as a fundamental structure that carries a constant positive charge. It was designed specifically for the separation of diastereomers of GSH, Cys, and Hcy enantiomers using UHPLC-Q-Orbitrap HRMS. The probe has a detection range of 2.40 to 7.20 fmol^30^. However, those above reagents such as DBD-pyNCS and GITC are not suitable for MS, but only for fluorescence or ultraviolet detection they failed to targeted identify amino or thiol functional groups without standards. Therefore, plenty of MS probes still need development to solve the current situation.

Given this, a novel MS probe Br-OTPP would be developed in this research. This study aims to establish a highly sensitive and selective UHPLC-HRMS method for simultaneous determination of seven types of thiols and tracing upstream differential proteins in PCNSL patients’ serum and cerebrospinal fluid based on labeled with Br-OTPP. Moreover, the feasible method is successfully applied to the authentic identification of PCNSL or control group by 3D PCA analyses. Besides, upstream proteins should be traced and thiol metabolic networks regulated by differential proteins maybe constructed. Ultimately, an intelligent monitoring model will be established to supervise PCSNL based on thiols related proteins joint machine learning.

## Results and discussion

### Synthesis of Br-OTPP

**Fig. 2:**
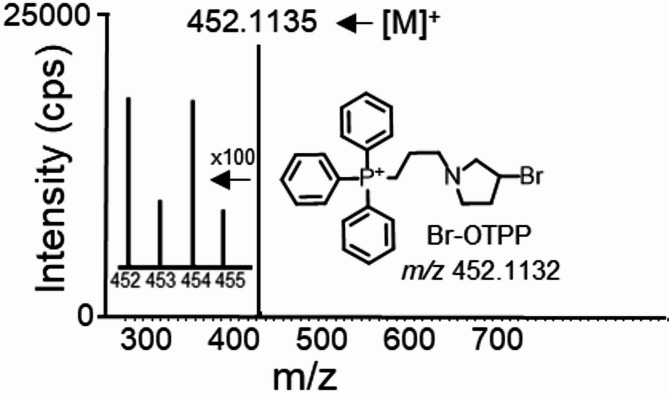
UHPLC-HRMS mass spectra and structure of mass spectrometry probe Br-OTPP. Br-OTPP: (3-(3-bromopyrrolidin-1-yl) propyl) triphenylphosphonium.

A method of labeling that utilizes a mass spectrometry probe with high selectivity and sensitivity is an efficient way to detect trace amounts of thiols in a C18 column for reversed-phase chromatography. In an earlier study, our team developed a novel chiral derivatization reagent called NCS-OTPP, which contains TPP as a basic structure with a permanent positive charge. This reagent was successfully employed to separate and detect thiols via LC-MS^30^. To label the thiol functional group, the isothiocyanate group of NCS-OTPP was replaced with a bromine group. A new mass spectrometry probe with a permanent positive charge structure was created for detecting thiols in serum and cerebrospinal fluid through a UHPLC-HRMS system. The structure of Br-OTPP was verified through instrumental analyses using NMR and HRMS. Notably, there were no interference peaks were observed in the UHPLC-HRMS mass spectra of Br-OTPP (as seen in Fig. 2). Additionally, this probe remained unchanged for 6 months at −20 ℃.

### Derivatization reaction of thiols with Br-OTPP

**Fig. 3:**
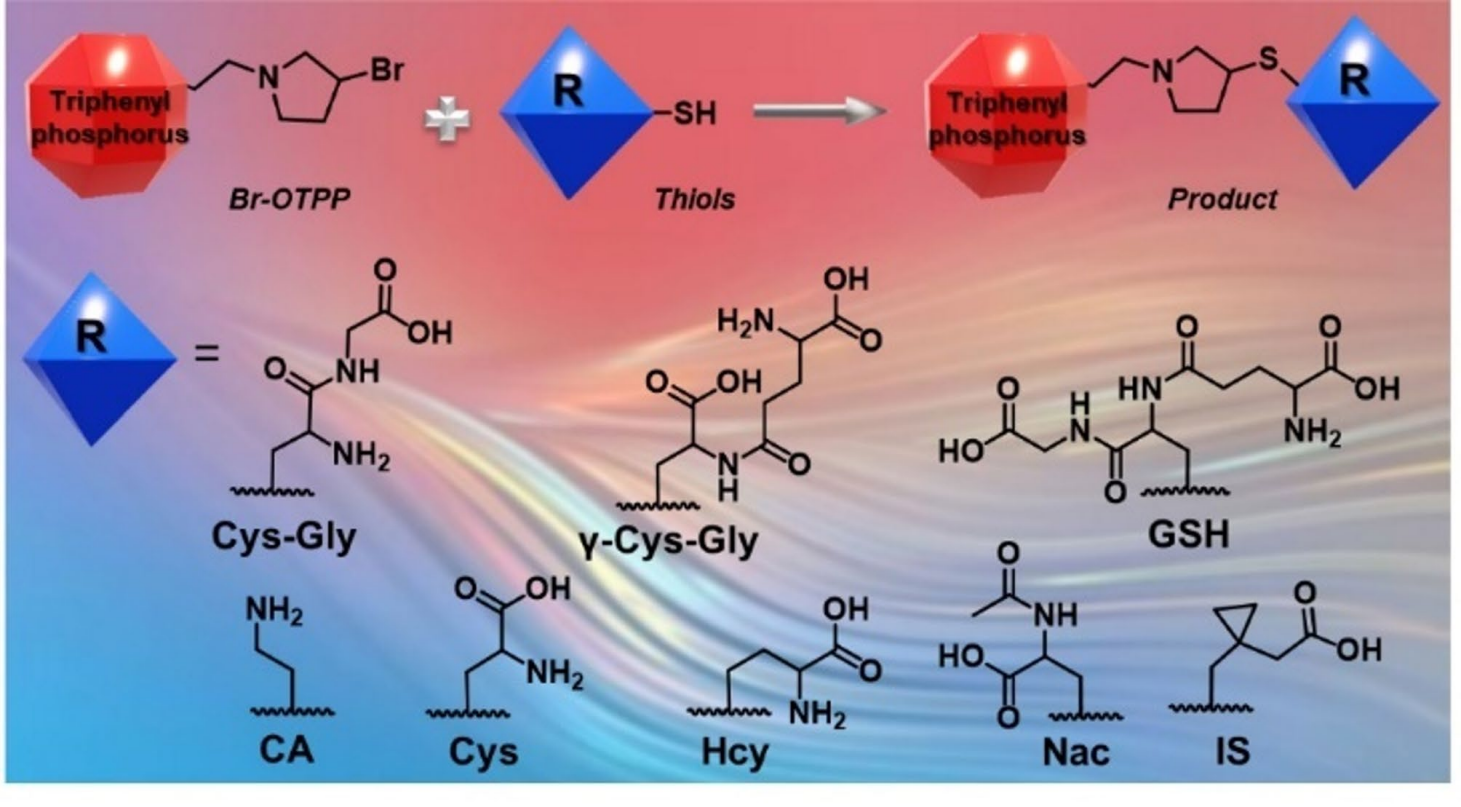
Profiling reaction and chemical structures of thiols with Br-OTPP.

**Fig. 4:**
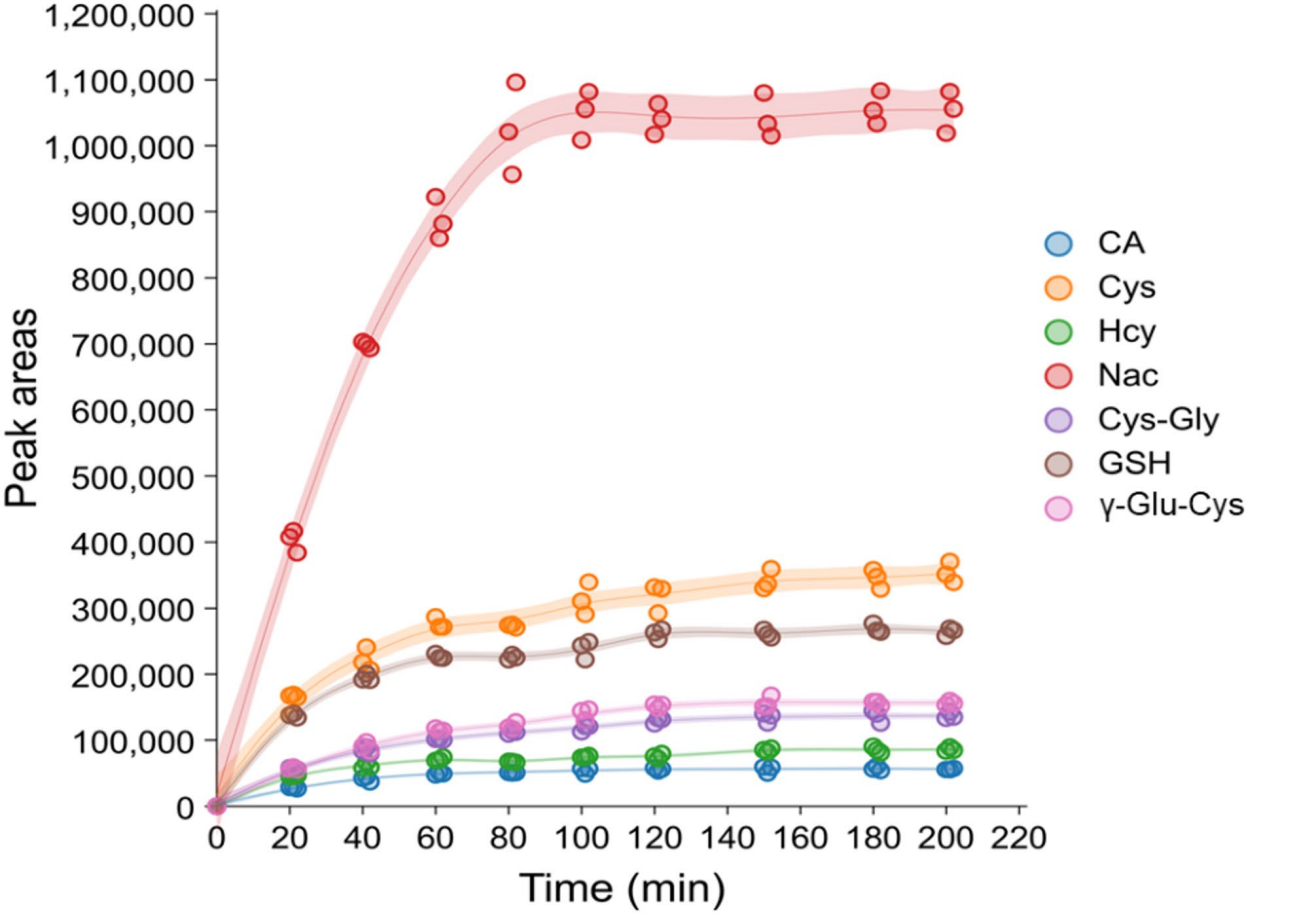
Time courses of the labelling reaction of thiols with Br-OTPP at 60 ℃.

The configurations and a modification process of thiols using Br-OTPP were examined are displayed in Fig. 3. The duration of the modification process was initially fine-tuned with indigenous thiols at 60 ℃, and later the quantities of modified products were scrutinized by UHPLC-HRMS. The progression of the labelling process of thiols with Br-OTPP is depicted in Fig. 4. The findings demonstrated that the magnitude of the peak area of Br-OTPP derivatives escalated progressively with the extension of the reaction period. Nevertheless, the peak area of the Br-OTPP derivatives tended to level off at 100 min and remained unvarying for 180 min. The course of labelling reaction was suitable for 7 kinds of thiols. Additionally, the patterns of the graphs obtained for the modifications of the other reagents were akin. Therefore, the most favorable reaction conditions for indigenous thiols were 60 ℃ or 100 min.

### Fragmentation pattern of thiol derives labeled by Br-OTPP on UHPLC-HRMS

**Fig. 5:**
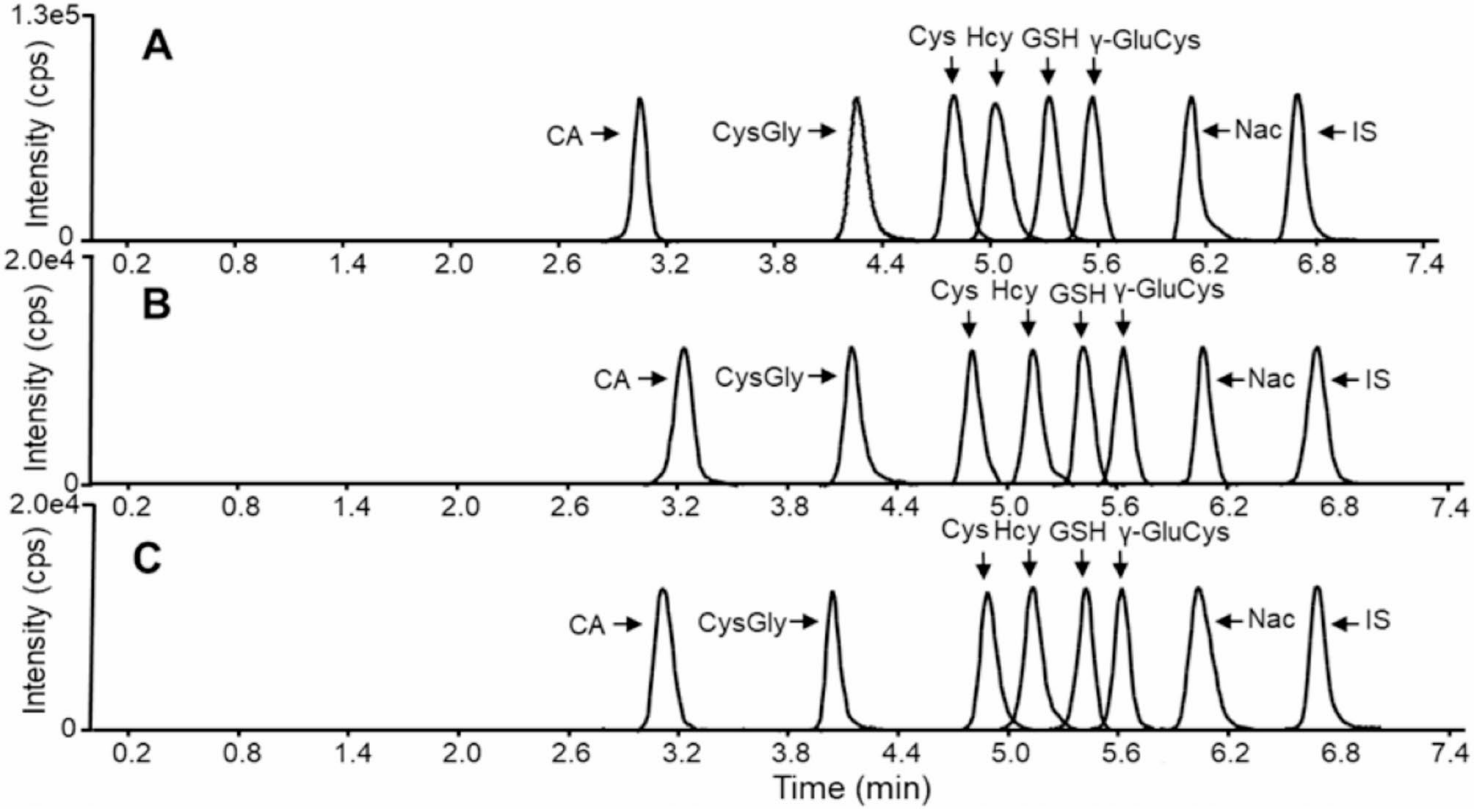
UHPLC-HRMS chromatograms of seven kinds of endogenous thiols in bio-samples from patients with primary central nervous system lymphoma. **A**: thiols standards; **B**: serum; **C**: cerebrospinal fluid.

**Fig. 6:**
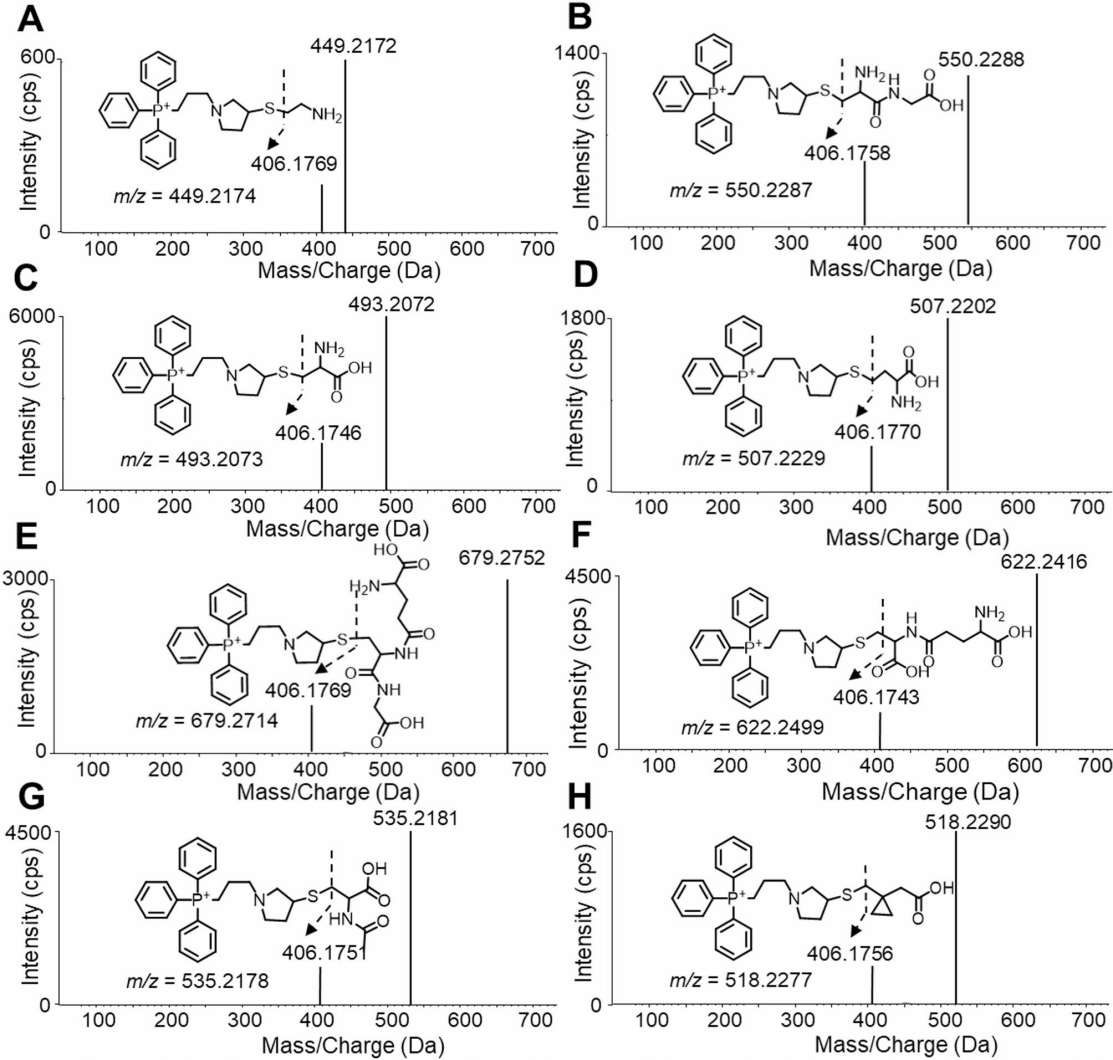
MS/MS product ion spectra and proposed fragmentation pattern with molecular formula and theoretical m/z of thiols derivative of Br-OTPP. **A**: CA; **B**: Cys-Gly; **C**: Cys; **D**: Hcy; **E**: GSH; **F**: γ-Glu-Cys; **G**: Nac; **H**: IS.

Considering that Br-OTPP can react with thiols and amino groups in alkaline environments, product ion scanning mode seems to be the preferred analytical strategy for thiol group target identification. The scan was performed in positive ion detection mode because Br-OTPP has a permanent positive charge, which improves the ionization efficiency in Fig. 5. Consequently, the thiol group underwent a qualitative examination by identifying its distinct fragment ions at the ideal collision energy. Fig. 6 illustrates that the thiol produced through Br-OTPP MS/MS spectrum labeling includes fragment ions at m/z 406.17, applicable for both qualitative and quantitative analysis. Crucially, our discovery revealed that the m/z 406.17 segment has the m/z 58.99 (R-C-S-R’) configuration instead of the m/z 28.02 (R-C-NH-R’) configuration, indicating Br -OTPP-labeled thiol-targeting characteristic ion as described in Table 1.

**Table 1:** IDA conditions of thiols labeled by Br-OTPP.

Thiols	Precursor ion (m/z)	Product ion (m/z)	Dell (s)	Cone voltage(v)	Collision Energy(v)
CA	449.21	406.17	0.25	20	49
Cys	493.22	406.17	0.25	20	50
Hcy	507.22	406.17	0.25	30	50
GSH	679.27	406.17	0.25	40	70
Nac	535.21	406.17	0.25	30	63
Cys-Gly	550.22	406.17	0.25	20	50
γ-Glu-Cys	622.25	406.17	0.25	40	68

### Validation of the proposed method

The calibration plots were generated using 7 distinct concentrations, with each concentration being repeated thrice. The calibration curves for every enantiomer of endogenous thiols were found to be satisfactory. Table 2 displays the equations of calibration curves and the limit of detection using the suggested process. The linear calibration curves obtained for each endogenous thiols were also excellent (R^2^ ≥ 0.9995). Additionally, the limit of detection (S/*N* = 3) ranged from 0.8 to 9.0 fmol, while the limit of quantification (S/*N* = 10) ranged from 8.0 to 12.0 fmol. The precision (CVs, %) of the present method was evaluated using intra-day and inter-day assays with three different concentrations. As indicated in Table S3, the CVs for intra-day and inter-day determinations ranged from 1.85 to 6.65% and 1.49–5.15%, respectively. A known concentration of QC samples was spiked into human serum to assess accuracy. Moreover Fig. 5 illustrates the UHPLC-HRMS chromatogram of 7 types of thiols in serum and cerebrospinal fluid of PCNSL patients (*n* = 30). Recovery was established by analyzing a spiked standard solution of known thiols concentration on the UHPLC-MS/MS system. Firstly, mean recoveries (%) of endogenous thiols spiked into serum ranged from 84.98 to 91.61%, as shown in Table 3. Furthermore, the CVs for intra-day and inter-day determinations were 0.99–5.54% and 1.53–5.84%, respectively. Similarly, Table 3 indicates that satisfactory accuracy of thiols spiked into cerebrospinal fluid was 85.28–97.88%. Moreover, CVs for intra-day and inter-day determinations were 1.58–5.53% and 2.08–5.49%. Because all analytes have good linearity, appropriate precision for intra-day and inter-day analysis, and satisfactory recovery accuracy, those requirements for serum and cerebrospinal fluid analysis can be met by this method.

**Table 2:** Calibration curves equations and limit of detection (LOD) of thiols using this method.

Thiols	Calibration range (pmol)	Linear equation	Linearity	LOD	LOQ
			(*R*^2^)	(fmol)	(fmol)
CA	0.024–250	y = 0.00007x − 0.0001	0.9995	8	12
Cys	0.024–500	y = 0.0022x + 0.00004	0.9999	7.5	12
Hcy	0.024–500	y = 0.00008x + 0.0003	0.9997	9	12
GSH	0.024–125	y = 0.0005x + 0.0004	0.9995	8	12
Nac	0.024–500	y = 0.0007x + 0.0013	0.9999	0.8	1.2
CysGly	0.024–250	y = 0.0019x − 0.0031	0.9999	7.5	12
γ-Glu-Cys	0.024–125	y = 0.0001x + 0.0001	0.9997	8	12

**Table 3:** Determination of thiols spiked in serum and cerebrospinal fluid of PCNSL patients.

Thiols	Spiked	Measured	Mean ± SD	Inter-day CV (%)		Intra-day CV (%)		Recovery		Mean Recovery	
	amont	Serum	/CSF	Serum	/CSF	Serum	/CSF	Serum	/CSF	Serum	/CSF
	(pmol)	(pmol)		(*n* = 6)		(*n* = 6)		(%)		(%)	
CA	0	0.05	0.03	3.37	2.62	3.48	1.63	-	-	88.83	93.15
	1.95	1.85 ± 0.03	1.93 ± 0.073	4.42	3.61	2.15	2.52	92.12	97.5		
	3.91	3.52 ± 0.12	3.64 ± 0.10	2.13	3.6	4.17	1.65	88.8	92.49		
	15.63	13.41 ± 0.15	14.00 ± 0.019	3.86	3.61	3.23	4.6	85.57	89.44		
Cys	0	244.62	1.68	3.92	3.08	2.34	3.68	-	-	87.09	93.65
	1.95	246.37 ± 0.60	3.59 ± 0.039	3.08	4.66	2.7	4.13	89.69	97.85		
	3.91	248.01 ± 5.13	5.34 ± 0.31	4.24	5.27	3.39	4.74	86.8	93.73		
	15.63	257.86 ± 4.65	15.65 ± 0.52	2.6	4.09	1.55	2.91	84.77	89.37		
Hcy	0	10.56	0.1	3.27	4.3	3.08	2.12	-	-	90.12	85.28
	1.95	12.39 ± 0.56	1.86 ± 0.03	3.18	5.45	1.46	3.69	93.78	89.95		
	3.91	14.04 ± 0.31	3.40 ± 0.08	2.94	4.11	3.59	5.07	89.14	84.48		
	15.63	24.22 ± 0.19	12.82 ± 0.17	3.63	2.49	2.81	3.87	87.44	81.4		
GSH	0	1.63	0.34	2.45	3.13	2.16	4.59	-	-	84.98	87.7
	1.95	3.35 ± 0.094	2.16 ± 0.11	5.58	4.53	2.83	4.05	88.28	93.14		
	3.91	4.94 ± 0.34	3.85 ± 0.31	4.17	2.17	4.21	4.22	84.66	89.79		
	15.63	14.44 ± 0.58	12.87 ± 0.086	1.59	2.32	3.46	4.58	82	80.17		
Nac	0	0.96	0.08	2.99	3.44	1.99	2.87	-	-	90.08	86.33
	1.95	2.79 ± 0.07	1.86 ± 0.045	4.69	2.56	0.99	4.31	94.06	91.18		
	3.91	4.57 ± 0.01	3.39 ± 0.10	3.95	4.1	3.38	3.73	92.53	84.84		
	15.63	14.07 ± 0.30	13.00 ± 0.32	4.99	3.37	4.23	3.71	83.65	82.98		
Cys-Gly	0	40.17	7.46	5.84	3.59	1.84	2.48	-	-	91.61	97.88
	1.95	42.05 ± 0.48	9.40 ± 0.57	5.2	3.21	1.86	2.74	96.05	99.42		
	3.91	43.82 ± 0.72	11.25 ± 0.50	3.85	3.86	3.95	2.97	93.37	97.31		
	15.63	53.52 ± 0.82	22.60 ± 1.26	4.07	3.28	3.8	2.18	85.41	96.91		
γ-Glu-Cys	0	5.56	0.22	4.02	2.08	2.22	1.58	-	-	88.48	86.86
	1.95	7.39 ± 0.27	1.98 ± 0.49	1.53	4.73	2.27	3.67	92.96	90.31		
	3.91	9.09 ± 0.22	3.67 ± 0.33	5.16	2.8	5.54	5.53	90.23	88.26		
	15.63	18.41 ± 0.51	13.03 ± 0.23	3.67	5.49	2.82	3.73	82.24	82.02		

### Determination of thiols related protein in PCNSL patients’ serum and cerebrospinal fluid

**Fig. 7:**
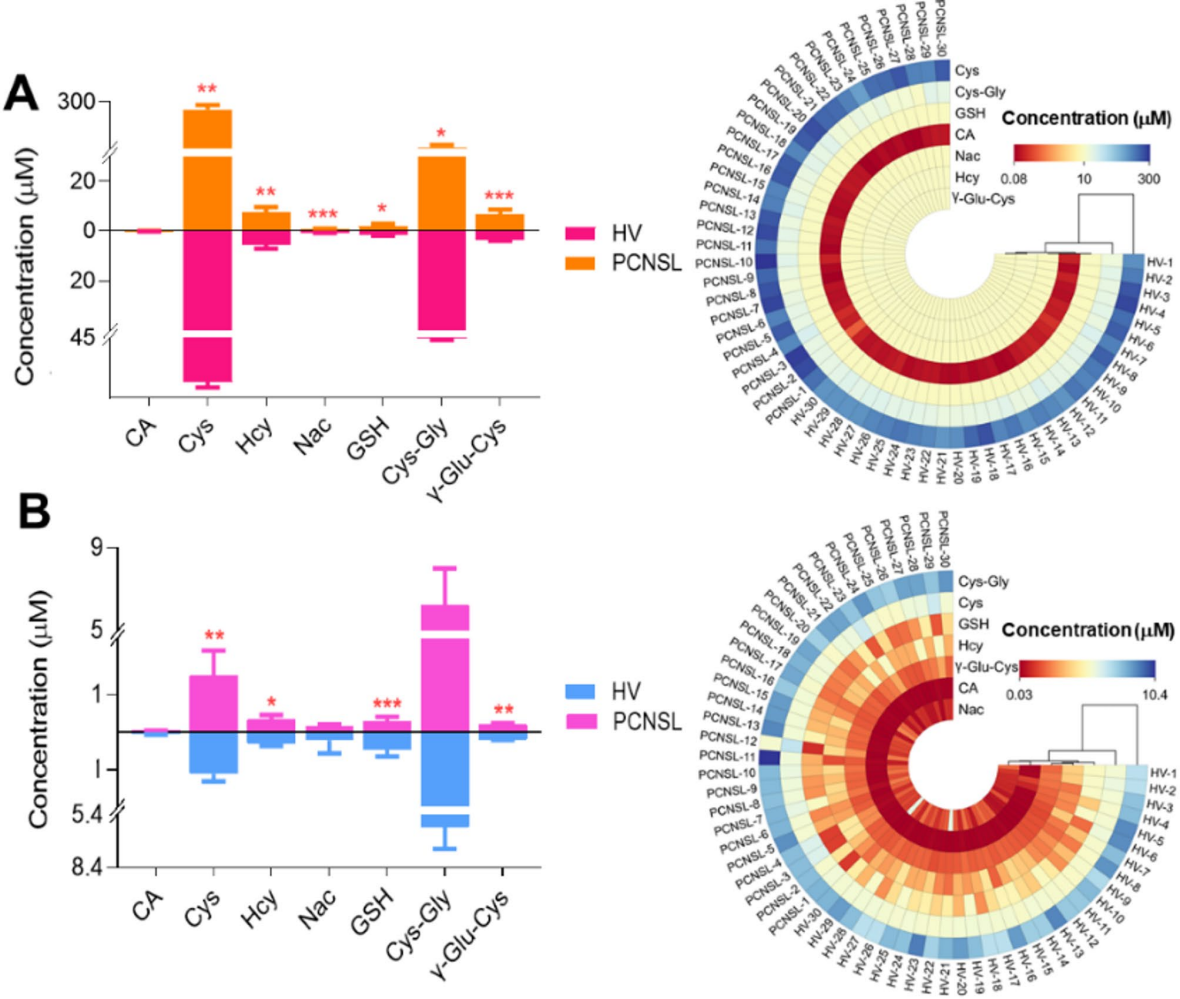
Determination of seven kinds of thiols in primary central nervous system lymphoma patients (PCNSL, *n* = 30) and healthy volunteer (HV, *n* = 30). (*p < 0.05, **p < 0.01, ***p < 0.001) **A**: Serum; **B**: cerebrospinal fluid.

**Fig. 8:**
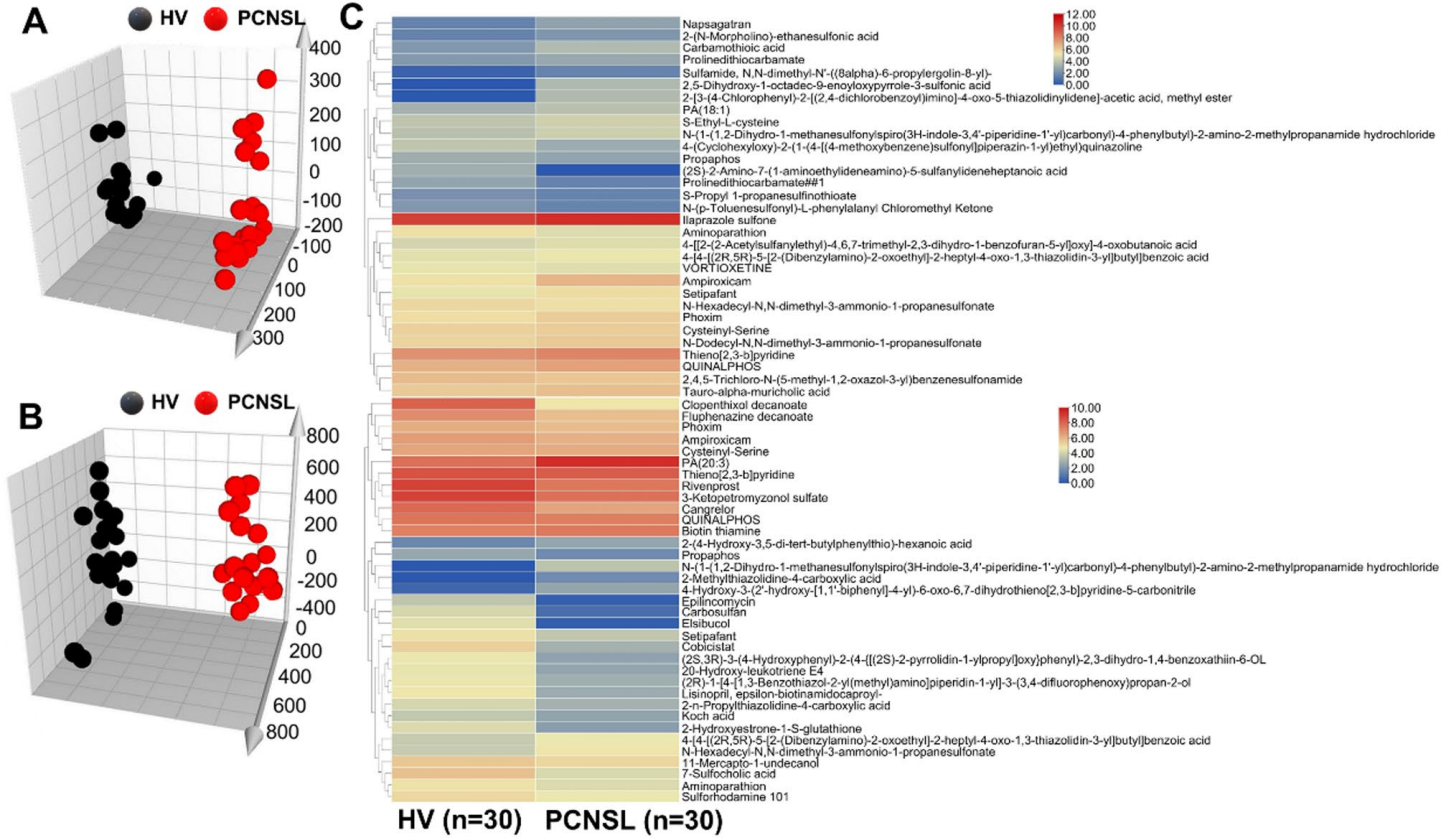
Multivariate statistics and cluster analysis of thiols in HV and PCNSL patients’ bio-samples. **A**: Serum; **B**: cerebrospinal fluid; **C**: Different thiol metabolites in PCNSL patients’ serum and cerebrospinal fluid.

**Fig. 9:**
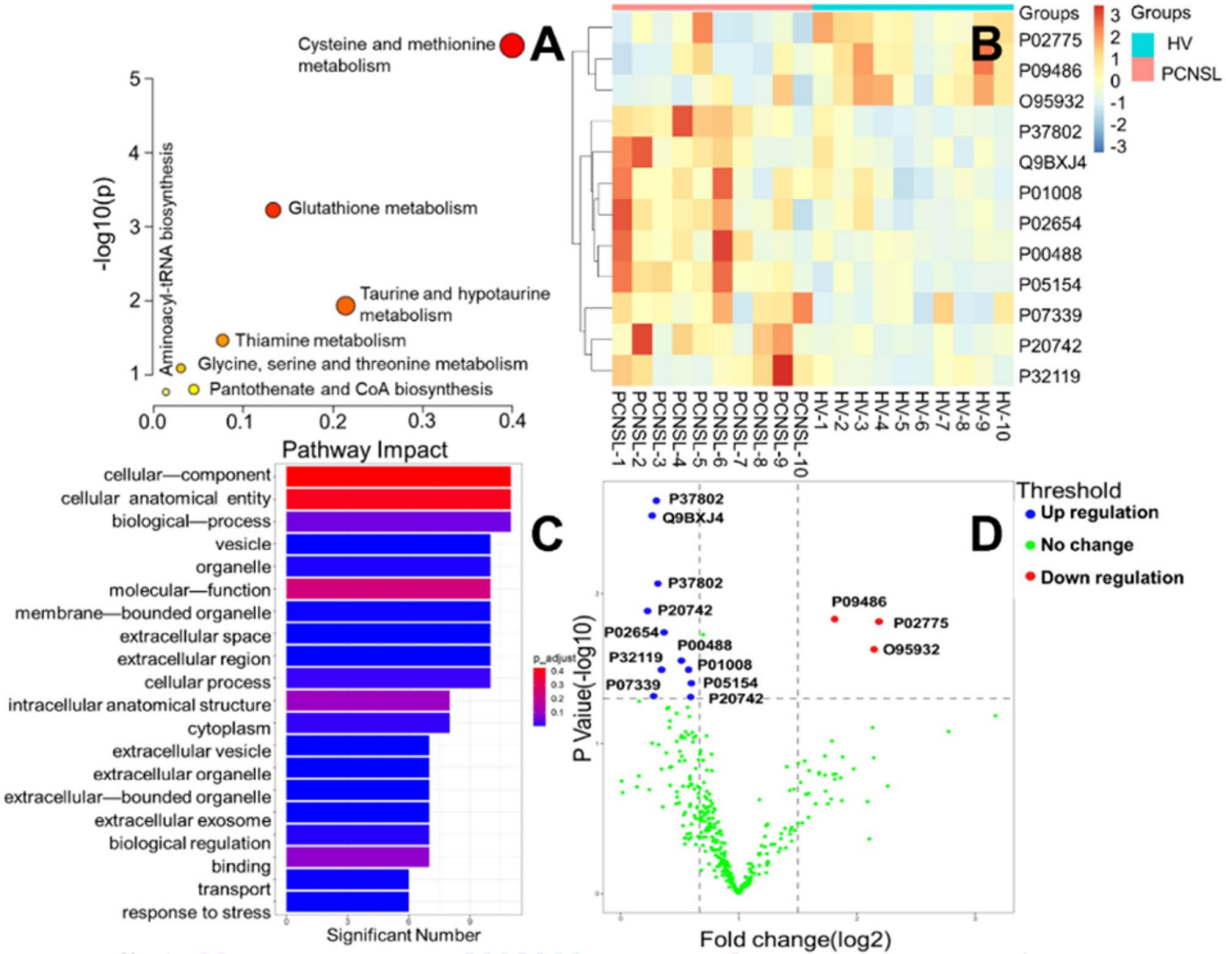
Determination of thiols related protein in serum of patients with primary central nervous system lymphoma (PCNSL, *n* = 10) and healthy volunteer (HV, *n* = 10). **A**: Metabolic pathway of thiol metabolites; **B**: heat map; **C**: GO bar plot; **D**: volcano map.

**Fig. 10:**
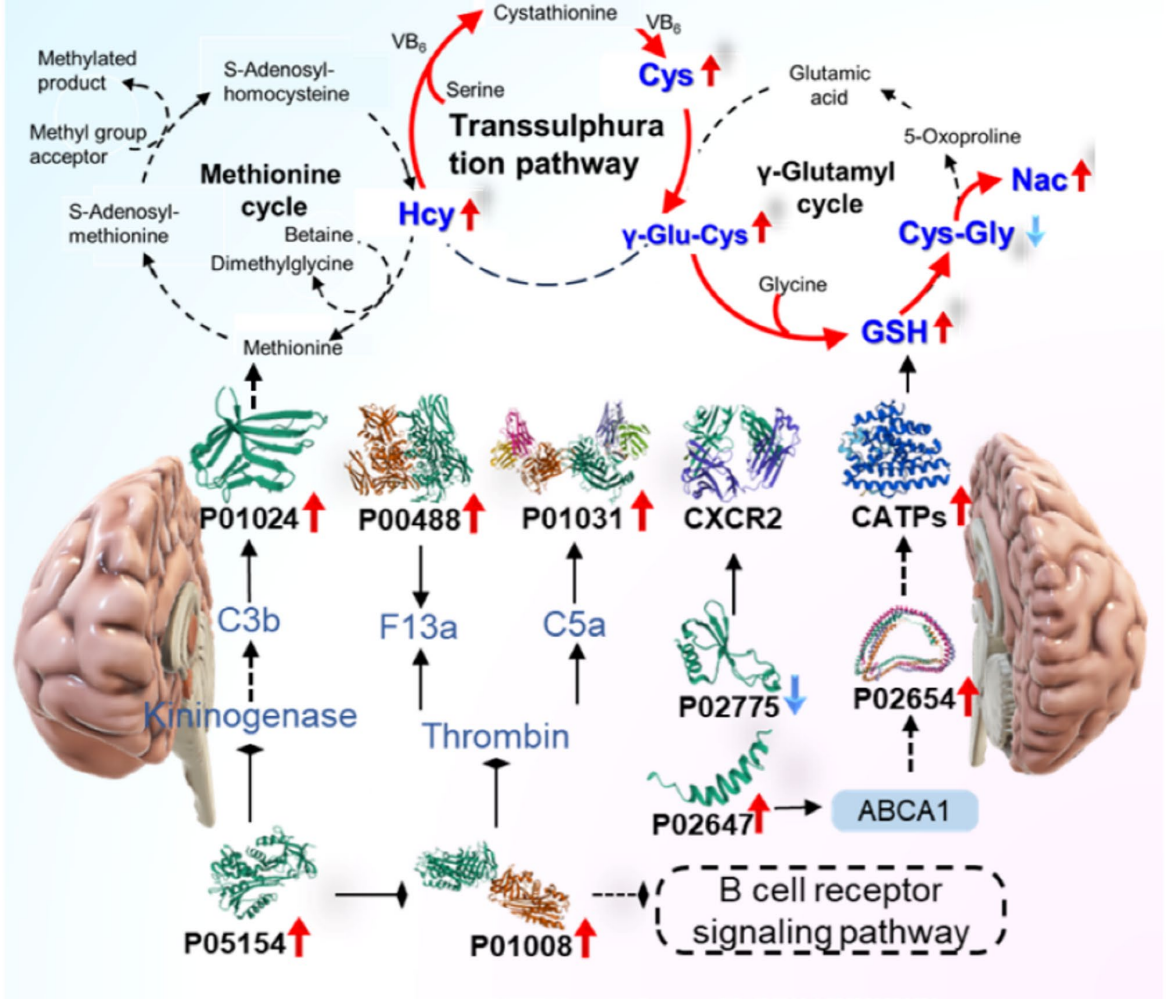
Thiol related proteins metabolic pathway in PCNSL patients.

The optimized and validated UHPLC-MS/MS method was applied to the analysis of thiols in bio-samples derived from 30 PCNSL patients and 30 healthy volunteers. The statistical analysis of 7 endogenous thiol concentrations in PCNSL’ serum and cerebrospinal fluid is shown in Fig. 7. We found out Cys, Hcy, Nac, GSH and γ-Glu-Cys levels of PCNSL patients’ serum were 1.089, 1.36, 1.25, 1.25 and 1.89 times higher than those in HV’ serum in Fig. 7A. While, CA and Cys-Gly contents of HV’ serum were 1.04 and 1.18 times greater than that in PCNSL patients (*p* < 0.05) in Fig. 7A. Figure 7B displayed Cys, Hcy, Cys-Gly and γ-Glu-Cys in cerebrospinal fluid from PCNSL patients were 1.36, 1.17, 1.02 and 1.09 times higher than that of HV’ cerebrospinal fluid. However, CA, Nac and GSH levels of HV were 1.14, 1.40 and 1.58 times than those in PCNSL patients (*p* < 0.05). Above all, the contents of Cys, Hcy, GSH, Cys-Gly and γ-Glu-Cys in serum and cerebrospinal fluid of PCNSL patients were significantly different from HV’ those. Furthermore, further verify the accuracy and reliability of those as PCNSL bio-markers, the proposed method was applied into distinction between PCNSL and control group in Fig. 8. We found out that thiols content in HV or PCNSL patients’ serum were distinguished for a specific 3D PLS-DA analysis as shown in Fig. 8A. Similarly, Fig. 8B described PCNSL and HV’ cerebrospinal fluid could achieve great separation. The results indicated that this method could pick out HV or PCNSL group by the difference of thiols in serum or cerebrospinal fluid. Moreover, Identification of thiol metabolites with characteristic fragment m/z = 406.17 after Br-OTPP labeled was performed by S-plot of thiol metabolites in HV and PCNSL patients’ serum and cerebrospinal fluid. Meanwhile, there are 62 and 51 kinds of different thiol metabolites in PCNSL and HV’ serum and cerebrospinal fluid, respectively. Heatmap of thiol metabolites further displayed the difference of their content distribution and those potential as PCNSL biomarkers in Fig. 8C. Also shown in Table 4 are 97 different sulfhydryl metabolites traced in the serum and CSF of PCNSL patients. Moreover Fig. 9 shows the determination of thiol-associated proteins in the serum of patients with primary central nervous system lymphoma (PCNSL, *n* = 10) and healthy volunteers (HV, *n* = 10). In addition, the thiols shown in Fig. 9A above may also be involved in the metabolism of glutathione, taurine and hypotaurine, cysteine and methionine, thiamine and glycine, serine and threonine, pantothenate and coenzyme a, and the biosynthesis of aminoacyl-trna. Subsequently, 340 proteins related to the above metabolic pathways were enriched and tracked, and Fig. 9B showed that the expression of 12 proteins was significantly different (*p* < 0.05). Fig. 9C shows the functional classification and biological processes of the related proteins. In addition, in Fig. 9D, SPARC (P09486), platelet basic protein (P02775), and protein-glutamine-γ-glutamyltransferase 6 (O95932) were significantly down-regulated, while all other proteins were up-regulated. Subsequently, we conducted a joint analysis of above proteins and thiols and found that the increase of plasma serine protease inhibitor (P05154) expression could negatively regulate the expression of antithrombin-III (P01008) and complement C3 (P01024), while the increase of antithrombin-III (P01008) expression could negatively regulate the expression of coagulation factor XIII A chain (P00488) and complement C5 (P01031). apolipoprotein A-I (P02647) would positively regulate the expression of apolipoprotein C-I (P02654) and CATPs. Significantly, CATPs and complement C3 (P01024) should directly act on GSH and methionine, promote the upregulation of Hcy in the methionine cycle, and indirectly cause the increase of Cys and γ-Glu-Cys on trans sulphuration pathway. Finally, thiol metabolic pathway develops towards γ-glutamyl cycle and causes up-regulation of Nac in Fig. 10.

**Table 4:** Different thiol metabolites in PCNSL patients’ serum and cerebrospinal fluid.

No.	Retention time (min)	Charge	m/z	Formula	Adducts	Mass Error (ppm)	Description	Serum	Cerebrospinal fluid
1	0.12	1	136.0209	C_7_H_5_NS	M + H	−4.83	Thieno(2,3)pyridine	*	*
2	3.83	1	637.3040	C_37_H_46_N_2_O_4_S	M + Na	−4.97	4-[4-[(2R,5R)−5-[2-(Dibenzylamino)−2-oxoethyl]−2-heptyl-4-oxo-1,3-thiazolidin-3-yl]butyl]benzoic acid	*	*
3	6.27	1	209.0596	C_6_H_12_N_2_O_4_S	M + H	2.45	Cysteinyl-Serine	*	*
4	0.28	1	284.0493	C_10_H_16_NO_3_PS	M + Na	4.69	Aminoparathion	*	*
5	0.40	1	299.0615	C_12_H_15_N_2_O_3_PS	M + H	0.54	Phoxim	*	*
6	1.04	1	299.0615	C_12_H_15_N_2_O_3_PS	M + H	0.36	Phoxim	*	*
7	1.04	1	299.0615	C_12_H_15_N_2_O_3_PS	M + H	0.36	Quinalphos	*	*
8	1.10	1	448.1177	C_20_H_21_N_3_O_7_S	M + H	0.98	Ampiroxicam	*	*
9	3.63	1	549.2512	C_28_H_38_N_4_O_4_S	M + Na	1.15	N-(1-(1,2-Dihydro-1-methanesulfonylspiro(3 H-indole-3,4’-piperidine-1’-yl)carbonyl)−4-phenylbutyl)−2-amino-2-methylpropanamide hydrochloride	*	*
10	8.79	1	414.2994	C_21_H_45_NO_3_S	M + Na	−4.74	N-Hexadecyl-N, N-dimethyl-3-ammonio-1-propanesulfonate	*	*
11	9.43	1	327.0780	C_13_H_21_O_4_PS	M + Na	−3.40	Propaphos	*	*
12	6.88	1	281.0808	C_17_H_12_O_4_	M + H, M + Na	−0.24	Mitoflaxone	*	*
13	9.43	1	327.0780	C_18_H_15_O_4_P	M + H	−0.21	Triphenyl phosphate	*	*
14	6.27	1	209.0596	C_14_H_8_O_2_	M + H	−0.69	1,2-Anthraquinone	*	*
15	6.27	1	209.0596	C_14_H_8_O_2_	M + H	−0.69	Anthraquinone	*	*
16	6.27	1	209.0596	C_14_H_8_O_2_	M + H	−0.69	9,10-Phenanthrenequinone	*	*
17	1.10	1	448.1177	C_23_H_20_FNO_6_	M + Na	2.47	((4-(3-(4-Fluoro-alpha-hydroxybenzyl)−4-hydroxyphenoxy)−3,5-dimethylphenyl)amino)oxoacetate	*	*
18	0.75	1	148.0425	C_5_H_9_NO_2_S	M + H	−1.44	2-Methylthiazolidine-4-carboxylic acid	*	
19	2.20	1	176.0740	C_7_H_13_NO_2_S	M + H	−0.01	2-n-Propylthiazolidine-4-carboxylic acid	*	
20	3.60	1	277.0681	C_10_H_16_N_2_O_3_S_2_	M + H	2.26	Biotin thiamine	*	
21	3.64	1	555.2808	C_32_H_43_ClN_2_O_2_S	M + H	0.24	Clopenthixol decanoate	*	
22	4.08	1	381.2212	C_20_H_32_N_2_O_3_S	M + H	1.41	Carbosulfan	*	
23	4.42	1	464.1907	C_27_H_29_NO_4_S	M + H	3.63	(2 S,3R)−3-(4-Hydroxyphenyl)−2-(4-{[(2 S)−2-pyrrolidin-1-ylpropyl]oxy}phenyl)−2,3-dihydro-1,4-benzoxathiin-6-ol	*	
24	5.50	1	478.2237	C_23_H_37_NO_6_S	M + Na	0.69	20-Hydroxy-leukotriene E4	*	
25	8.32	1	797.9364	C_17_H_25_Cl_2_F_3_N_5_O_12_P_3_S_2_	M + Na	−1.47	Cangrelor	*	
26	9.69	1	489.2520	C_24_H_40_O_8_S	M + H	0.64	7-Sulfocholic acid	*	
27	9.93	1	614.2125	C_28_H_37_N_3_O_9_S	M + Na	−3.08	2-Hydroxyestrone-1-S-glutathione	*	
28	9.93	1	904.4776	C_46_H_76_NO_11_PS	M + Na	−3.08	PA(20:3(5Z,8Z,11Z)/LTE4)	*	
29	0.67	1	541.1201	C_26_H_23_ClN_6_O_2_S	M + Na	−3.08	Setipafant	*	
30	3.19	1	451.2149	C_24_H_34_O_6_S	M + H	−3.08	Rivenprost	*	
31	3.33	1	495.2410	C_24_H_40_O_7_S	M + Na	−3.08	3-Ketopetromyzonol sulfate	*	
32	4.34	1	405.9324	C_10_H_9_NO_9_S_3_	M + Na	−3.08	Koch acid	*	
33	4.43	1	614.2989	C_32_H_44_F_3_N_3_O_2_S	M + Na	−3.08	Fluphenazine decanoate	*	
34	5.39	1	625.3346	C_35_H_54_O_4_S_2_	M + Na	−3.08	Elsibucol	*	
35	5.42	1	205.1618	C_11_H_24_OS	M + H	−3.08	11-Mercapto-1-undecanol	*	
36	5.43	1	361.0637	C_20_H_12_N_2_O_3_S	M + H	−3.08	4-Hydroxy-3-(2’-hydroxy-[1,1’-biphenyl]−4-yl)−6-oxo-6,7-dihydrothieno[2,3-b]pyridine-5-carbonitrile	*	
37	5.61	1	407.2196	C_18_H_34_N_2_O_6_S	M + H	−3.08	Epilincomycin	*	
38	6.39	1	629.1388	C_31_H_30_N_2_O_7_S_2_	M + Na	−3.08	Sulforhodamine 101	*	
39	8.15	1	434.1693	C_22_H_25_F_2_N_3_O_2_S	M + H	−3.08	(2R)−1-[4-[1,3-Benzothiazol-2-yl(methyl)amino]piperidin-1-yl]−3-(3,4-difluorophenoxy)propan-2-ol	*	
40	8.35	1	745.3956	C_37_H_56_N_6_O_8_S	M + H	−3.08	Lisinopril, epsilon-biotinamidocaproyl-	*	
41	9.29	1	353.2128	C_20_H_32_O_3_S	M + H	−3.08	2-(4-Hydroxy-3,5-di-tert-butylphenylthio)-hexanoic acid	*	
42	9.69	1	798.3433	C_40_H_53_N_7_O_5_S_2_	M + Na	−3.08	Cobicistat	*	
43	3.33	1	495.2410	C_21_H_36_N_4_O_8_	M + Na	−3.08	(2R,4 S)−4-Carbamimidamido-3-acetamido-2-((1R,2R)−2-hydroxy-1-methoxy-3-(octanoyloxy)propyl)−3,4-dihydro-2 H-pyran-6-carboxylic acid	*	
44	3.68	1	509.2560	C_31_H_32_N_4_O_3_	M + H	−3.08	1-[[4-(Dimethylamino)−3-methylphenyl]methyl]−5-(2,2-diphenylacetyl)−6,7-dihydro-4 H-imidazo[4,5-c]pyridine-6-carboxylic acid	*	
45	3.90	1	249.0434	C_15_H_11_Cl	M + Na	−3.08	9-(Chloromethyl)anthracene	*	
46	4.13	1	412.2490	C_25_H_33_NO_4_	M + H	−3.08	Etorphine	*	
47	4.27	1	301.0591	C_16_H_10_N_2_O_3_	M + Na	−3.08	Dehydroxymethylflazine	*	
48	4.42	1	464.1907	C_24_H_25_N_5_O_5_	M + H	−3.08	1-Methyl-5-(4-benzoyl)pyrrole-2-acetic acid 2-(theophylline-7-yl)ethyl ester	*	
49	4.55	1	413.1267	C_14_H_22_N_4_O_9_	M + Na	−3.08	Epitalon	*	
50	4.55	1	413.1267	C_22_H_21_ClN_2_O_4_	M + H	−3.08	PYRAOXYSTROBIN	*	
51	5.39	1	625.3346	C_32_H_44_N_6_O_7_	M + H	−3.08	Cyclo(D-Trp-D-Asp-Pro-D-Ile-Leu)	*	
52	8.15	1	434.1693	C_23_H_21_N_7_O	M + Na	−3.08	Entospletinib	*	
53	8.15	1	434.1693	C_24_H_23_N_3_O_5_	M + H	−3.08	4-Nitrophenyl 4-(3-phenoxybenzyl)piperazine-1-carboxylate	*	
54	8.15	1	434.1693	C_22_H_22_F_3_N_3_O_3_	M + H	−3.08	1-(2-Methyl-4-methoxyphenyl)−4-((2-hydroxyethyl)amino)−6-trifluoromethoxy-2,3-dihydropyrrolo(3,2-c)quinoline	*	
55	8.84	1	386.2322	C_23_H_31_NO_4_	M + H	−3.08	Stachyflin	*	
56	9.29	1	353.2128	C_23_H_28_O_3_	M + H	−3.08	11,17-Dihydroxy-6-methyl-17-(1-propynyl)androsta-1,4,6-triene-3-one	*	
57	5.61	1	407.2196	C_24_H_32_O_4_	M + Na	−3.08	7-[(1 S,2R,3R,4R)−3-(3-Hydroxy-4-phenylpent-1-enyl)−7-oxabicyclo[2.2.1]heptan-2-yl]hept-5-enoic acid	*	
58	5.61	1	407.2196	C_24_H_32_O_4_	M + Na	−3.08	Estradiol dipropionate	*	
59	5.61	1	407.2196	C_24_H_32_O_4_	M + Na	−3.08	Magestin	*	
60	3.90	1	251.9852	C_6_H_3_N_3_O_7_	M + Na	−3.08	Picric acid	*	
61	3.19	1	451.2149	C_24_H_31_FO_7_	M + H	−3.08	Androsta-1,4-diene-17-carboxylicacid, 17-[(ethoxycarbonyl)oxy]−11-hydroxy-3-oxo-, fluoromethyl ester, (11b,17a)-	*	
62	3.19	1	451.2149	C_25_H_27_FN_4_O_3_	M + H	−3.08	Cediranib	*	
63	0.51	1	167.0551	C_6_H_14_OS_2_	M + H	−3.08	S-Propyl 1-propanesulfinothioate		*
64	2.29	1	134.0629	C_5_H_11_NOS	M + H	−3.08	Carbamothioic acid		*
65	2.29	1	134.0629	C_5_H_11_NOS	M + H	−3.08	methyl 4-mercaptobutyrimidate		*
66	2.29	1	134.0629	C_5_H_11_NOS	M + H	−3.08	2-Amino-4-methylsulfanylbutanal		*
67	3.22	1	460.2381	C_22_H_37_NO_7_S	M + H	−3.08	2,5-Dihydroxy-1-octadec-9-enoyloxypyrrole-3-sulfonic acid		*
68	4.57	1	880.4755	C_44_H_76_NO_11_PS	M + Na	−3.08	PA(18:1(11Z)/LTE4)		*
69	7.55	1	148.0245	C_5_H_9_NS_2_	M + H	−3.08	Prolinedithiocarbamate		*
70	0.52	1	340.9315	C_10_H_7_Cl_3_N_2_O_3_S	M + H	−3.08	2,4,5-Trichloro-N-(5-methyl-1,2-oxazol-3-yl)benzenesulfonamide		*
71	0.53	1	541.1195	C_26_H_23_ClN_6_O_2_S	M + Na	−3.08	Setipafant		*
72	0.58	1	383.1156	C_19_H_18_N_4_O_3_S	M + H	−3.08	Ilaprazole sulfone		*
73	0.75	1	150.0578	C_5_H_11_NO_2_S	M + H, M + Na	−3.08	S-Ethyl-L-cysteine		*
74	3.24	1	516.3015	C_26_H_45_NO_7_S	M + H	−3.08	2-[(3alpha,7alpha,12alpha-Trihydroxy-24-oxocholane-24-yl)amino]ethanesulfonic acid		*
75	3.24	1	516.3015	C_26_H_45_NO_7_S	M + H	−3.08	Tauro-alpha-muricholic acid		*
76	3.99	1	533.2191	C_27_H_34_N_4_O_4_S	M + Na	−3.08	4-(Cyclohexyloxy)−2-(1-(4-[(4-methoxybenzene)sulfonyl]piperazin-1-yl)ethyl)quinazoline		*
77	4.14	1	405.2325	C_21_H_32_N_4_O_2_S	M + H	−3.08	Sulfamide, N,N-dimethyl-N’-((8alpha)−6-propylergolin-8-yl)-		*
78	4.19	1	321.1401	C_18_H_22_N_2_S	M + Na	−3.08	VORTIOXETINE		*
79	4.19	1	468.9592	C_19_H_11_Cl_3_N_2_O_4_S	M + H	−3.08	2-[3-(4-Chlorophenyl)−2-[(2,4-dichlorobenzoyl)imino]−4-oxo-5-thiazolidinylidene]-acetic acid, methyl ester		*
80	6.03	1	403.1180	C_19_H_24_O_6_S	M + H, M + Na	−3.08	4-[[2-(2-Acetylsulfanylethyl)−4,6,7-trimethyl-2,3-dihydro-1-benzofuran-5-yl]oxy]−4-oxobutanoic acid		*
81	6.88	1	148.0244	C_5_H_9_NS_2_	M + H	−3.08	Prolinedithiocarbamate		*
82	6.88	1	148.0244	C_5_H_9_NS_2_	M + H	−3.08	Pyrrolidine dithiocarbamate		*
83	6.88	1	581.2145	C_26_H_34_N_6_O_6_S	M + Na	−3.08	Napsagatran		*
84	7.09	1	196.0636	C_6_H_13_NO_4_S	M + H	−3.08	2-(N-Morpholino)-ethanesulfonic acid		*
85	7.18	1	380.0714	C_18_H_18_ClNO_4_S	M + H	−3.08	Methyl 2-(2-acetoxy-6,7-dihydrothieno[3,2-c]pyridin-5(4 H)-yl)−2-(2-chlorophenyl)acetate		*
86	7.18	1	380.0714	C_18_H_18_ClNO_4_S	M + H	−3.08	N-(p-Toluenesulfonyl)-L-phenylalanyl Chloromethyl Ketone		*
87	8.51	1	358.2374	C_17_H_37_NO_3_S	M + Na	−3.08	N-Dodecyl-N, N-dimethyl-3-ammonio-1-propanesulfonate		*
88	8.67	1	254.0925	C_9_H_17_N_3_O_2_S	M + Na	−3.08	(2 S)−2-Amino-7-(1-aminoethylideneamino)−5-sulfanylideneheptanoic acid		*
89	3.29	1	216.0665	C_9_H_10_FNO_4_	M + H	−3.08	2-Amino-3-(3-fluoro-4,5-dihydroxyphenyl)propanoic acid		*
90	3.29	1	216.0665	C_9_H_10_FNO_4_	M + H	−3.08	2-Fluoro-5-hydroxy-L-tyrosine		*
91	3.29	1	216.0665	C_9_H_10_FNO_4_	M + H	−3.08	2-Amino-3-(2-fluoro-3,4-dihydroxyphenyl)propanoic acid		*
92	3.29	1	216.0665	C_12_H_9_NO_3_	M + H	−3.08	6-Oxo-1-phenyl-1,6-dihydropyridine-3-carboxylic acid		*
93	6.88	1	148.0244	C_4_H_5_NO_5_	M + H	−3.08	N-Oxalylglycine		*
94	6.88	1	581.2145	C_29_H_30_N_6_O_6_	M + Na	−3.08	Olmesartan medoxomil		*
95	7.55	1	148.0245	C_4_H_5_NO_5_	M + H	−3.08	N-Oxalylglycine		*
96	8.51	1	358.2374	C_22_H_31_NO_3_	M + H	−3.08	4,17-Dimethyltrilostane		*
97	8.51	1	358.2374	C_22_H_31_NO_3_	M + H	−3.08	Phencynonate		*

### Intelligent monitoring PCNSL patients based on thiols associated proteins with machine learning

**Fig. 11:**
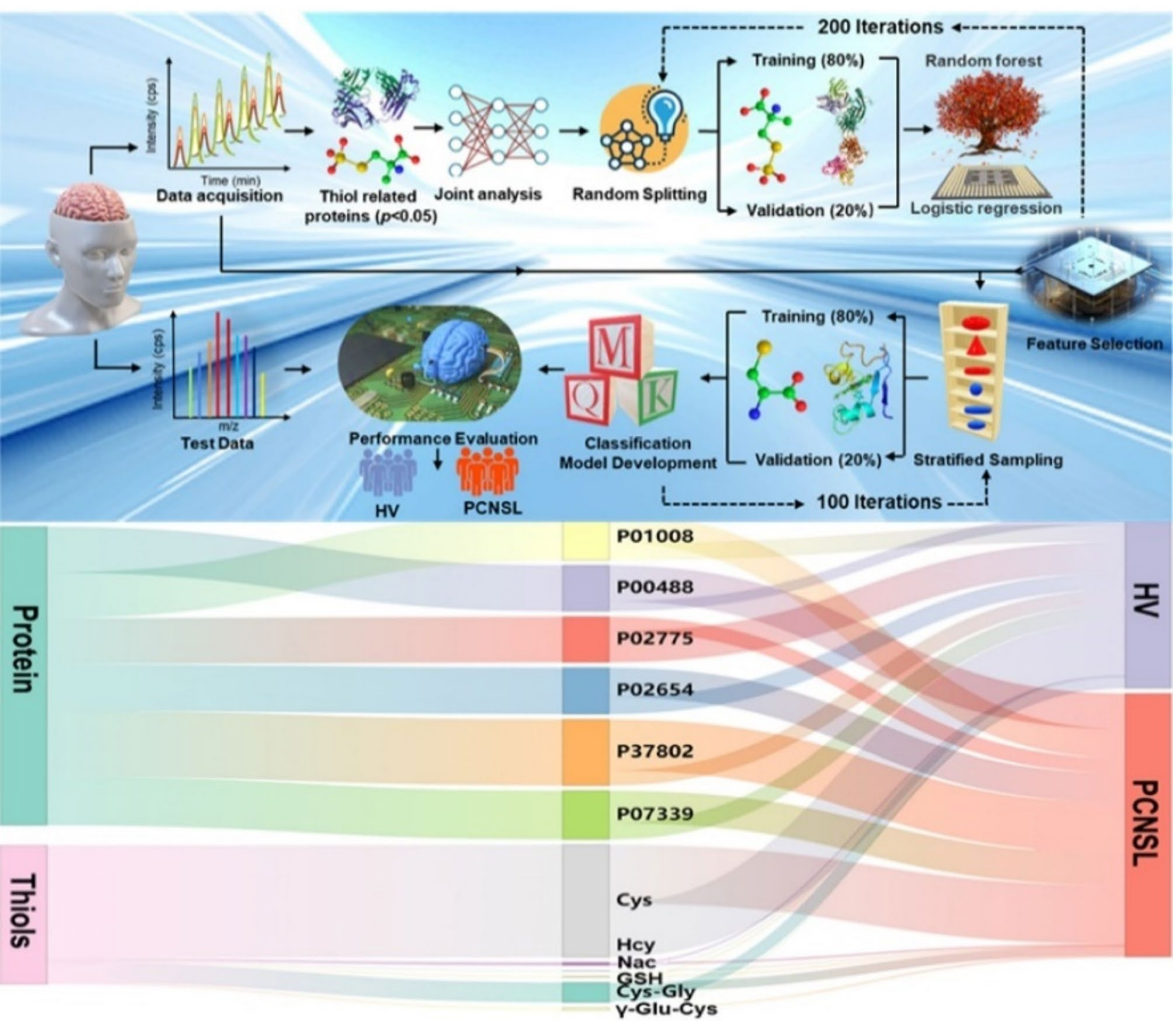
Performance of the random forests and logistic regression-based machine learning model.

We independently performed ML on the entire dataset of 11 thiol related proteins obtained from 10 PCNSL patients and 10 HVs to construct a classification model based on random forest and logistic regression analysis for predicting PCNSL and identifying the most important features of classification in Table S1. Set-I (the learning dataset) contains 80% of the stratified samples to build the ML model and find the feature im-portance score, while Set-II (the hold Set) contains the remaining 20% to independently test the model’s performance in Fig. 11. Fourteen were randomly selected as the training set and the remaining six as the test set, and 200 trials were conducted with an average readiness of 98.83% in Table S2. These results highlight the robustness of thiol associated proteins as potential diagnostic biomarkers for PCNSL to achieve high accuracy in distinguishing cancer from normal groups.

## Materials and methods

### Materials

Br-OTPP (Synthesized in the laboratory), (3-Bromopropyl) triphenyl phosphonium bromide (Br-TPP), 3-Bromopyrrolidine hydrobromide, triethylamine (TEA), Glutathione (GSH), Cysteinyl-glycine (Cys-Gly), γ-Glutamyl-cysteine (γ-Glu-Cys), and N-Acetyl-L-cysteine (Nac), Tris (2-carboxyethyl) phosphine hydrochloride (TCEP) and 1-(mercaptomethyl)cyclopro paneacetic acid were purchased from Aladdin Corpora-tion (USA). Cysteine (Cys), Homocysteine (Hcy), Cysteamine (CA) were bought from Sigma-Aldrich (USA), Methanol (MeOH), acetonitrile (ACN), Ethylenediaminetetraacetic acid (EDTA), Formic acid (FA), and trifluoroacetic acid (TFA) were of MS re-agent grade (Fisher, USA). Every other chemical classified as analytical reagent grade was used without further purification. Deionized and distilled water (H2O) (Unique-R20 Multifunctional Ultrapure Water System, Research Scientific Instruments Co., Xiamen, China) was used throughout the study.

### UHPLC triple TOF MS conditions

The ultra-high performance liquid chromatography system linked with a Triple TOF 5600^+^ (AB Sciex, USA) was utilized for the UHPLC Triple TOF high-resolution mass spectrometry analysis. Chromatographic separation was carried out on a Kinetex C18 (2.0 × 100 mm, 1.7 μm) column at 40 ℃. Seven varieties of thiols were separated through gradient elution using 0.1% formic acid in water as mobile phase A and 0.1% formic acid in acetonitrile as mobile phase B. The entire analysis process was concluded within 0–1-4–5-6–8 min by performing gradient elution of 8–13-13–20-30–40% mobile phase B. The automatic sampler was prepared at 4 ℃. The electrospray ionization source is used by the mass spectrometer and the quality test is performed in the positive ion mode shown in Table 5. 50 L/min was the ion source gas flow setting value, 30 L/min was the curtain gas flow setting value, the ion spray floating voltage was 5.5 kV, and the source temperature was 500 ℃. Ions in the range of 50 ~ 1000 m/z with the resolution of 100,000 FWHM were used to scan data using the IDA mode. SCIEX OS software 2.0.3 was used to obtain the mass spectra of target analytes. The SCIEX OS-MQ software was utilized to import and process all MS information and data. This enabled the acquisition of precise mass measurements for product ions to be used in IDA and Product ion mode. The chosen mode for product ion selection was based on selected ion scanning of precursor ions, the NCE is specified in the inclusion list. The key to achieve the highest response of chromatographic peak is the best NCE selection of product ions. To ensure reliability and accuracy, accurate mass measurements based on molecular ions are both qualitative and quantitative results with a mass error of less than 1 ppm. Seven types of product ions were monitored using IDA for each derivative: CA-Br-OTPP m/z 449.2172→406.1769; Cys-Gly-Br-OTPP m/z 550.2288→406.1758; Cys-Br-OTPP m/z 493.2072→406.1746; Hcy-Br-OTPP m/z 507.2202→406.1770; GSH-NCS-OTPP m/z 679.2752→406.1769; γ-Glu-Cys m/z 622.2416→406.1743 and Nac m/z 535.2181→406.1751 were monitored. These productions were utilized for qualitative and quantitative analysis owing to the labeling of thiol group characteristic ion fragments and increased signal intensity Table 6.

**Table 5:** UHPLC-HRMS conditions.

UHPLC (Exion, AB SCIEX, USA)	
Column	Kinetex C18 (2.0 × 100 mm, 1.7 μm)
Mobile phase A	0.1% FA in H_2_O
Mobile phase B	0.1% FA in CH_3_CN
Gradient elution	8–13-13–20-30–40% (0–1-4–5-6–8 min)
Column Temperature	40 ℃
Flow rate	0.40 mL/min
Injection volume	1 µL
Q-TOF HRMS (Triple TOF 5600 + MS, AB SCIEX, USA)	
Polarity	ESI+
Declustering potential	60 V
Ion release delay	67 V
Ion release width	25 V
Source temperature	500 ℃
Ion source gas	50 L/min
Curtain gas	30 L/min
MS range	50–1000 *m/z*
Orbitrap Eclipse Tribrid HRMS (Thermo Fisher Scientific, USA)	
Ion Source Type	NSI
Expected LC Peak Width	30 s
Positive Ion	2100 V
Ion Transfer Tube Temp	400 ℃
Orbitrap Resolution	1,000,000
Scan Range	350–2000 *m/z*

**Table 6:** Full name and abbreviation.

ABBREVIATION	FULL NAME
PCNSL	primary central nervous system lymphoma patients
UHPLC-HRMS	Ultra-high-performance liquid chromatography-high resolution mass spectrometry
MS	Mass spectrometry
NGS	Next-generation sequencing
UHPLC	Ultra-high performance liquid chromatography
HPLC-UV	High performance liquid chromatography-ultraviolet
HPLC-FL	High performance liquid chromatography-fluorescence
3D PCA	3D principal component analysis
DBD-PyNCS	4-(3-isothiocyanatopyrrolidin-1-yl)−7-(N, N-dimethylaminosulfonyl)−2,1,3-benzoxadiazole
OPA	o-phthalaldehyde
GITC	2,3,4,6-tetra-O-acetyl-β-D-glucopyranosyl isothiocyanate
NCS-OTPP	(R)-(5-(3-isothiocyanatopyrrolidin-1-yl)−5-oxopentyl) tri-phenylphosphonium
TPP	triphenylphosphine
Br-OTPP	(3-(3-bromopyrrolidin-1-yl) propyl) triphenylphosphonium
IDA	information dependent acquisition
NCE	normalized collision energy
LOD	Limit of detection
LOQ	Limit of quantitation
ML	machine learning
FAIMS	High-field asymmetric waveform ion mobility spectrometry
mL	Milliliters
℃	Celsius degrees
mg	Milligrams

### Synthesis of Br-OTPP for thiols mass derivatization probe

A suitable quantity of (3-bromopropyl) triphenylphosphonium bromide and 3-bromopyrroli-dine hydrobromide were included in a dehydrated round-bottomed flask and dissolved using N, N-dimethylformamide. Next, anhydrous potassium car-bonate was introduced and the mixture was allowed to react at normal temperature for a duration of 6 h. The resulting solution was extracted with water and dichloromethane thrice. The Br-OTPP novel thiol mass spectrometry probe was obtained by evaporating the dichloromethane layer. HRMS: m/z 452.1135 [M]^+^. 1 H NMR (400 MHz, CDCl_3_) δ: 7.85–7.60 (m, 15 H), 4.35–4.29 (m, 1 H), 3.99–3.75 (m, 2 H), 2.94–2.65 (m, 6 H), 2.38 (dddd, J = 15.7, 14.0, 11.3, 7.2 Hz, 2 H), 1.92–1.72 (m, 2 H).

### Preparation and derivative reaction of thiols

A blend of 0.77 mg of CA, 1.78 mg of Cys-Gly, 1.21 mg of Cys, 1.35 mg of Hcy, 3.07 mg of GSH, 2.50 mg of γ-Glu-Cys and 1.63 mg of Nac was measured and dissolved in a mixture of a solution of ace-tonitrile-water (1:1, volume/volume). The internal standard (IS) substance, 1.46 mg of 1-(mercaptomethyl)cyclopropaneacetic acid, was weighed and diluted to 1 millimolar concentration. Then, 50 µL of thiols and 50 µL of IS were added to a tube, followed by the sequential addition of 50 µL of 5% triethylamine and 50 µL of 5 mM Br-OTPP. The tube was placed in a constant-heat oscillator with 1000 rpm at 60 ℃, and a 1.0 µL sample was injected into the ultra-high-performance liquid chromatography-tandem mass spectrometry system.

### PCNSL patients’ serum and cerebrospinal fluid sample collection and pretreatment

Thiols detection was carried out using serum and cerebrospinal fluid samples obtained from 30 HV and 30 patients diagnosed with PCNSL. All volunteers were in good physical condition, non-smokers and non-drinkers. The samples were collected without any analyte supplementation. Prior to collection, the volunteers were given informed consent forms to read and sign voluntarily. This work was approved by the biomedical research ethics committee of Shandong provincial hospital affiliated to Shandong First Medical University (NO.2024 − 795). All studies were conducted in accordance with relevant guidelines/regulations and informed consent was obtained from all participants. Blood samples were collected from HV and PCNSL patients who were in a fasting state using medical vacuum tubes containing separation gel and coagulant. The tubes were then centrifuged at 1500 rpm for 15 min at 4 ℃ to obtain serum and cerebrospinal fluid. The supernatant was stored in small tubes with rubber stopper screw caps, with each tube holding 100 µL volume of serum or cerebrospinal fluid. The supernatant was either immediately subjected to detection or stored at −80 ℃ until analysis. The serum pretreatment process was slightly modified based on published literature^30^. A mixture of 50 µL ACN, 50 µL of 10 mM EDTA, and 10 µL of 20 mM TCEP was added to the tubes containing 100 µL bio-samples and 50 µL of 1 mM IS, and incubated for 10 min at 25 ℃. The tubes were then subjected to deproteinization by adding 140 µL ACN and centrifuging at 10,000 g at 4 ℃ for 15 min. The resulting supernatant solution (100 µL) was transferred to a new tube, followed by the addition of 50 µL of 5% TEA and 50 µL of 5 µM Br-OTPP in sequence. The tube was then incubated in a constant-heat oscillator at 60 ℃ for 100 min.

### Validation of the method

Seven sets of calibration curves were created using varying levels of thiols found in serum or cerebrospinal fluid. Each thiols (CA, Cys-Gly, Cys, Hcy, GSH, γ-Glu-Cys and Nac) had linear calibration curves consisting of seven points (thiol peak area/IS peak area vs. thiol concentration). The calibration points were measured three times within the range of 0.12–250 pmol. LOD and LOQ were ascertained through a signal-to-noise (S/N) ratio of 3:1 and 10:1, in that order. The precision and accuracy both within and between days were ascertained through the analysis of six quality control specimens. The accuracy (measured as coefficient of variation, CV%) of each concentration was determined using six repeated measurements. The evaluation of analytical recovery involved examining six duplicated spiked serum samples, with quality assurance samples created by applying spiking standards at varying concentrations (1.95, 3.91, and 15.63 µM for thiols, respectively) to three distinct serum or CSF samples. The recovery percentage was ascertained by contrasting the reactions of samples that were spiked with those that were not.

### Determination of thiols related proteins in PCNSL patients’ serum and cerebrospinal fluid

 The changes of thiols and related different proteins in serum and cerebrospinal fluid of PCNSL patients were investigated during therapeutic session. Moreover, sample collection was performed under the condition of nothing supplies were provided for HV and PCNSL patients when they entered the hospital. Furthermore, each other’s blood pressure, heart rates and physical health index were measured. On one hand, the content of endogenous thiols was collected and determined by lumbar puncture and venous blood as described in Sect. 3.5. On the other hand, as for proteins, 10 µL plasma was added into abundant protein reagent and centrifuged at 1000 g and 4 ℃ for 2 min to extract protein. Moreover, 10 µL protein, 110 µL PBS and 120 µL BCA working liquid were placed in incubator at 37 ℃ for 105 min. Take 100 µg protein, fill UA Buffer into 250 µL ultrafiltration tube, centrifuge at 13,600 rpm and 4 ^o^C for 20 min, repeat twice. Then 200 µL DTT was added and incubated at 37 ℃ for 1.5 h, centrifuged at 16,000 rpm for 20 min, then 50 mM IAA 200 µL was added, reaction was carried out at room temperature away from light for 30 min, centrifuged at 16,000 rpm for 20 min, the filtrate was discarded and the operation was repeated twice. Then 0.25 µg/uL pancreatic enzyme 8 µL and 100 µL ammonium bicarbonate were added, incubated overnight at 37 ℃, centrifuged at 12,000 rpm for 20 min, 50 µL ammonium bicarbonate was added and centrifuged again at room temperature, and then the bottom cap of the desalt column was placed into 2 mL tube at 5000 g for 1 min, and the filtrate was discarded. The column was placed into a 2 ml tube, 300 µL 0.1% TFA-50% acetonitrile was added, centrifuged at 3000 g for 1 min, filtrate was collected and transferred to a 1.5 mL tube, and the operation was repeated. A total of 600 µL filtrate was collected and frozen and concentrated before injection.

### Establishment of machine learning model and data processing and analysis

In this work, ML is based on thiol related proteins data independently based on two cohorts (PCNSL and HV). Stratified sampling was used to randomly divide the samples into two groups. The first group (Set-I) consisted of 20 samples (10 normal samples and 10 PCNSL samples) and was used to identify the corresponding thiol associated protein (*p* < 0.05) and determine its importance score using random forest and logistic regression analysis algorithms. Then the maximum likelihood classification model is constructed by using the random forest and logistic regression supervised learning algorithm. The second group, consisting of 6 samples (3 normal samples and 3 cancer samples), was retained as a holding trial group to evaluate the performance of the final ML model. The ML model is trained on 20 samples randomly drawn from the Set-I and verified on the remaining 6 samples. The final model was tested on 20 maintenance samples from Set-II. Moreover, data acquisition and processing were carried out with SCIEX OS and SCIEX OS-MQ software (AB SCIEX, USA), respectively. The statistical analyses with a Student’s t-test used with IBM SPSS Statistics 21.0. Results of (* *p* < 0.05), (** *p* < 0.01) and (*** *p* < 0.001) were considered significant. The line chart and bar chart whisker-box plot used with GraphPad Prism 9.0.

## Conclusions

In summary, a novel mass spectrometry probe Br-OTPP was developed for the targeted identification of thiol metabolites. A highly sensitive and selective UHPLC-HRMS method was established for simultaneous quantitative determination of 7 kinds of thiols and tracing 97 types of different thiol metabolites in serum and cerebrospinal fluid of PCNSL patients. Furthermore, Upregulated complement C3 (P01024) and CATPs directly positively regulated methionine and GSH, further promoted others increase on thiol metabolic pathway by FAIMS tandem Orbitrap Eclipse. Ultimately, a highly accurate PCNSL monitoring model were developed for the first time based on thiols associated proteins combined with machine learning algorithm. In short, the intelligent analysis strategy is demonstrated to be a promising tool to study the thiols and different proteins occurring in transsulphuration pathway, methionine and γ-glutamyl cycle, which offers the opportunity for indepth investigation of the pathogenesis and prognostic monitoring of PCNSL. Further determinations of many patient’s serum and cerebrospinal fluid samples are currently underway in our laboratory.

## Electronic supplementary material

Below is the link to the electronic supplementary material.

Supplementary Material 1

## Data Availability

Data is provided within the manuscript or supplementary information files.

## Reprints and permissions information

is available at www.nature.com/reprints.
