# Supplementary material for: Simultaneous determination of 7 thiols associated proteins in lymphoma patients’serum and cerebrospinal fluid by UHPLC-HRMS technique
Source: Sci Rep. 2025 Jul 3;15:23783. doi: 10.1038/s41598-025-03023-6 (PMC12229638; doi:10.1038/s41598-025-03023-6)
Supplement: Supplementary file 1 — Supplementary Material 1 [file 41598_2025_3023_MOESM1_ESM.docx]

Table S1. Survival data of thiols associated proteins.

| No. | P-value | Fold Change |
| --- | --- | --- |
| P01008 | 0.04 | 0.61 |
| P00488 | 0.05 | 0.28 |
| P02775 | 0.02 | 2.13 |
| P02654 | 0.03 | 0.58 |
| P37802 | 0.00 | 0.30 |
| P07339 | 0.03 | 0.51 |
| Cys | ≤0.01 | 1.08 |
| Hcy | ≤0.01 | 1.35 |
| Nac | ≤0.001 | 1.30 |
| GSH | ≤0.05 | 1.32 |
| Cys-Gly | ≤0.05 | 0.96 |
| γ-Glu-Cys | ≤0.001 | 1.96 |

Table S2. Machine learning parameters based on thiols associated proteins.

| Set. | Pathology | Predicted | | Accuracy | Mean Accuracy |
| --- | --- | --- | --- | --- | --- |
|  |  | HV | PCNSL | (%) | (%) |
| Training | HV | 7 | 0 | 100 | 98.21% |
|  | PCNSL | 0 | 7 | 100 |  |
| Test | HV | 3 | 0 | 100 | 98.58% |
|  | PCNSL | 0 | 3 | 100 |  |
| All | HV | 10 | 0 | 100 | 98.83% |
|  | PCNSL | 0 | 10 | 100 |  |

Table S3. Accuracy and precision of this method by intra-day and inter-day assays.

| Thiols (μM) | | Intra-day (n=6） | | | Inter-day (n=6) | | |
| --- | --- | --- | --- | --- | --- | --- | --- |
|  |  | Mean (μM) | RSD (%) | Accuracy (%) | Mean (μM) | RSD (%) | Accuracy (%) |
| CA | 1.95 | 1.97 | 4.12 | 100.78 | 1.88 | 1.61 | 96.03 |
|  | 15.63 | 14.39 | 3.82 | 92.1 | 13.97 | 3.06 | 89.38 |
|  | 31.25 | 31.65 | 2.67 | 101.29 | 26.75 | 5.15 | 85.6 |
| Cys | 1.95 | 2.02 | 2.98 | 103.28 | 2 | 3.52 | 102.61 |
|  | 15.63 | 13.79 | 2.57 | 88.28 | 13.3 | 2.62 | 85.11 |
|  | 31.25 | 27.92 | 3.64 | 89.34 | 27.4 | 1.49 | 87.7 |
| Hcy | 1.95 | 2.04 | 2.7 | 104.57 | 2.05 | 3.76 | 104.76 |
|  | 15.63 | 15.74 | 2.83 | 100.71 | 15.84 | 5.02 | 101.41 |
|  | 31.25 | 30.19 | 6.65 | 96.61 | 29.92 | 2.55 | 95.76 |
| GSH | 1.95 | 1.87 | 6.01 | 95.88 | 1.88 | 2.8 | 96.16 |
|  | 15.63 | 14.11 | 1.85 | 90.28 | 13.54 | 3.1 | 86.65 |
|  | 31.25 | 27.06 | 2.26 | 86.59 | 25.38 | 3.27 | 81.23 |
| Nac | 1.95 | 2.04 | 3.8 | 104.24 | 1.92 | 2.87 | 98.13 |
|  | 15.63 | 15.62 | 2.87 | 99.95 | 14.25 | 2.55 | 91.19 |
|  | 31.25 | 29.22 | 2.48 | 93.52 | 27.25 | 2.67 | 87.21 |
| Cys-Gly | 3.9 | 4.02 | 4.66 | 103.03 | 3.99 | 4.4 | 102.24 |
|  | 15.63 | 15.1 | 6.56 | 96.63 | 15.23 | 3.09 | 97.5 |
|  | 125 | 116.75 | 3.8 | 93.4 | 120.49 | 1.91 | 96.39 |
| γ-Glu-Cys | 1.95 | 2.91 | 2.91 | 87.78 | 1.98 | 2.23 | 101.23 |
|  | 15.63 | 3.63 | 5.92 | 101.75 | 15.6 | 2.77 | 99.86 |
|  | 31.25 | 3.13 | 3.13 | 102.94 | 30.3 | 3.06 | 96.96 |
